# Increased LOH Due to Defective Sister Chromatid Cohesion Is Due Primarily to Chromosomal Aneuploidy and Not Recombination

**DOI:** 10.1534/g3.117.300091

**Published:** 2017-10-03

**Authors:** Dror Sagi, Evgeniya Marcos-Hadad, Vinay K. Bari, Michael A. Resnick, Shay Covo

**Affiliations:** * Department of Plant Pathology and Microbiology, Hebrew University, Rehovot 76100, Israel; † Chromosome Stability Group, Laboratory or Molecular Genetics, National Institute of Environmental Health Sciences, NIH, Research Triangle Park, North Carolina 27709

## Abstract

Loss of heterozygosity (LOH) is an important factor in cancer, pathogenic fungi, and adaptation to changing environments. The sister chromatid cohesion process (SCC) suppresses aneuploidy and therefore whole chromosome LOH. SCC is also important to channel recombinational repair to sister chromatids, thereby preventing LOH mediated by allelic recombination. There is, however, insufficient information about the relative roles that the SCC pathway plays in the different modes of LOH. Here, we found that the cohesin mutation *mcd1-1*, and other mutations in SCC, differentially affect the various types of LOH. The greatest effect, by three orders of magnitude, was on whole chromosome loss (CL). In contrast, there was little increase in recombination-mediated LOH, even for telomeric markers. Some of the LOH events that were increased by SCC mutations were complex, *i.e.*, they were the result of several chromosome transactions. Although these events were independent of *POL32*, the most parsimonious way to explain the formation of at least some of them was break-induced replication through the centromere. Interestingly, the *mcd1-1 pol32*Δ double mutant showed a significant reduction in the rate of CL in comparison with the *mcd1-1* single mutant. Our results show that defects in SCC allow the formation of complex LOH events that, in turn, can promote drug or pesticide resistance in diploid microbes that are pathogenic to humans or plants.

Sister chromatid cohesion (SCC) assures proper chromatid segregation by tethering newly replicated sister chromatids until mitosis (Onn *et al.* 2008; Xiong and Gerton 2010). In yeast, SCC is primarily accomplished by the four subunit cohesin complex containing Smc1, Smc3, Mcd1, and Scc3. Defects in SCC can lead to aneuploidy, and are associated with several developmental defects (Bose and Gerton 2010) and cancer (Yamamoto *et al.* 2006; Barber *et al.* 2008; Xu *et al.* 2011).

Cohesin is specifically enriched around the centromeres. This is due, in part, to the protein Mcm21 that facilitates SCC around the centromere in a manner yet to be determined (Ortiz *et al.* 1999; Poddar *et al.* 1999; Ng *et al.* 2009). The centromere enrichment of cohesin facilitates sister chromatid biorientation before mitosis (Ng *et al.* 2009; Stephens *et al.* 2011, 2013), assuring proper chromatid segregation, and the prevention of aneuploidy. Binding of cohesin to chromatin is not enough for SCC. In order to establish SCC, the cohesin complex is activated during DNA replication (Lengronne *et al.* 2006). Establishment of SCC in S phase is primarily regulated by Eco1-dependent acetylation ( Rolef Ben-Shahar *et al.* 2008; Unal *et al.* 2008; Ivanov *et al.* 2002; Zhang *et al.* 2008; Rowland *et al.* 2009; Skibbens 2009, and references therein). One of the roles of Eco1 is to counteract the antiestablishment activity of Wpl1. Wpl1 appears to prevent the establishment of SCC at G2 (Guacci and Koshland 2012; Lopez-Serra *et al.* 2013), but it is important for maintenance of SCC once it is properly established (Rolef Ben-Shahar *et al.* 2008; Rowland *et al.* 2009; Sutani *et al.* 2009).

On top of its role in chromosome segregation, cohesin also facilitates DNA double-strand break (DSB) repair between sister chromatids, and suppresses recombination between homologous chromosomes (Sjogren and Nasmyth 2001; Covo *et al.* 2010; Heidinger-Pauli *et al.* 2010). Thus, cohesin is supposed to increase genome stability through prevention of recombination-generated loss of heterozygosity (LOH), as well as preventing chromosome segregation errors that could also lead to LOH. While SCC can influence recombination, its effect on various types of recombination or LOH has not been addressed. To the best of our knowledge, the only report about change in the nature of recombination events in relation to SCC comes from studies with an *eco1* mutant that exhibits lower cross-over frequency (Lu *et al.* 2010).

Interhomolog-recombination, in mitotic cells, may result in several outcomes. Most frequently, recombination occurs through gene conversion (GC) nonassociated with cross-over adjacent to the site of a DSB. The information exchanged between the two chromosomes is limited and local. To a lesser extent, GC events that are associated with reciprocal or nonreciprocal cross-over occur. Recombination can also occur through establishment of an alternative replication fork following a DSB—known as Break Induced Replication (BIR) (Ira *et al.* 2003; Haber *et al.* 2004; Jain *et al.* 2009). BIR is often dependent on the DNA polymerase *δ* accessory subunit Pol32. Both cross-over and BIR involve significant exchange of information between the two chromosomes; the information exchange is not confined to a locus but usually involves segments of the chromosomes (Haber *et al.* 2004; Deem *et al.* 2008). Numerous genetic assays to study LOH and other recombination events were previously developed; some of them were designed to differentiate between alternative recombination outcomes (Lee *et al.* 2009; Ho *et al.* 2010; Symington and Gautier 2011; St Charles *et al.* 2012). We employed here a genetic assay to study LOH; however, our assay aims only to differentiate between local recombination events and segmental ones.

We show that, in yeast, cohesin primarily suppresses LOH through preventing chromosome loss (CL). Then cohesin suppresses complex LOH events. Sister chromatid cohesion has very little effect on segmental and local recombination events in our experimental system. We propose that the genome plasticity of diploid cells defective in SCC may facilitate adaptive evolution such as that which occurs in pathogenic fungi and cancer cells (Tischfield 1997; Forche *et al.* 2008; Lamour *et al.* 2012).

## Materials and Methods

### Strain construction

Gene inactivation was done by knock-out of specific open reading frames using the KanMX cassette from the *Saccharomyces cerevisiae* deletion collection. The primers that were used for *WPL1* knock-out were 1 and 2 (all primers are shown in Supplemental Material, Table S2 in File S1); the primers for *MCM21* knockout were 3 and 4; the primers for deleting *RAD51* were 5, 6; the primers used to delete *POL32* were 7, 8. *mcd1-1* strains were created by pop-in/pop-out of pVG257 (Guacci *et al.* 1997), and the mutation was verified by sequencing. Haploid strains that were used to construct the loss of heterozygosity strains were described in Covo *et al.* (2014a). Briefly, the haploid strains were transformed with the selectable markers (NAT^R^, *URA3*, HygB^R^, and *TRP1*) using PCR products with the following primers. Insertion of NAT cassette was done using primers 9 and 10, pAG25 served as a template. Validation was done by primers 11 and 12. Insertion of *URA3* cassette next to the centromere was done by using primers 13 and 14 using pRS306 as a template. Validation was done by using primers 15 and 16. Insertion of the Hyg resistance cassette was done using primers 17 and 18; pAG32 was used as a template. Validation was done by using primers 19 and 20. Insertion of *TRP1* cassette was done using primers 21 and 22. Validation was done using primers 23 and 24.

In order to create an *ura3nonsense* mutant next to the centromere, a *URA3* cassette was introduced at position 241,000 in chromosome II (see Figure 2) in strain CS1120 using primers 13, 14. Then, a spontaneous 5FOA-resistant colony was isolated and sequenced. The mutation was identified to be a G→T change that created a stop codon at the third codon. This strain (CS 1294) was crossed with a *mcd1-1* strain that contains all ectopic markers on chromosomes II (CS 1234) as shown in Figure 2. Creation of wild type (WT) and *mcd1-1* with a telomeric *URA3* marker was done by introducing the *URA3* gene to NAT+ HYG+ TRP+ strains from the respective backgrounds using primers 25 and 26 with pRS306 as a template. Validation was done using primers 27 and 28.

Diploid strains to detect LOH were created by mating two opposite mating-type haploids, which, in this background, carry a different mutation in the methionine biosynthesis pathway (*met2Δ* and *met6Δ*). Diploid cells were selected on methionine-less plates and verified for all other genetic markers. Molecular genotyping of 5FOA resistant strains was done with the primers that were used for validation of insertion of NAT, HYG, *TRP1*, and *URA3* cassettes and primers 29 and 30 for *TYR1*.

### Determination of the pattern of LOH in different genetic backgrounds

Diluted liquid cultures (100 cells/µl) were plated by a 121-prong device to YPDA plates to create 121 mini-cultures (∼10^6^ cells/mini-culture) [for more details see Jin *et al.* (2003) and references therein]. Cells were then replica-plated to 5FOA plates. From each mini-culture, a single 5FOA resistant colony was picked and further analyzed. The 5FOA-resistant colonies were spotted onto YPDA plates and then replica-plated to YPDA plates containing HYG and NAT, and to synthetic complete plates lacking tryptophan. The full phenotype of each independent 5FOA colony was then determined.

### Rate determination for each LOH scenario

General conditions for rate determination of LOH are described in the following paragraph. Experiments were started by patching at least six Uracil prototroph single colonies of each genotype to YPDA rich medium, followed by incubation overnight at 30°, including *mcd1-1* temperature-sensitive strains (*mcd1-1* strains are grown and maintained at 23° prior to the experiment). Overnight patches were then spread on selective media (5FOA) without dilution (WT and *wpl1Δ*), or with 10- to 100-fold dilution (*mcm21Δ* and *mcd1-1*). All genotypes were diluted 100,000 or 50,000 and spread on synthetic complete medium to determine the culture size. To restrict the effect of *mcd1-1* mutation to the growth phase and not to the selection phase, plates were incubated at 23°. Plates were incubated for 2–4 d. The number of colonies grown on complete and 5FOA media was in the range of 50–500 depending on the genotype and dilutions.

All 5FOA resistant colonies from each patch were spotted to YPDA plates and then analyzed for retention of HYG, NAT and TRP markers as described above. In order to determine the rate of complex events in *mcd1-1* strains, undiluted cultures were spread on 5FOA plates. The lawn of yeast was replica-plated to NAT plates or –TRP plates. Next, NAT+ ura– hyg– trp– or TRP+ hyg–, ura– nat– colonies were counted. The rate (micro) of each event was calculated according to Drakes “Method of Median” (Drake 1991), with modifications as described in Spell and Jinks-Robertson (2004) and references therein. The median and 95% C.I. were calculated according to (Massey and Dixon 1969).

### Pulsed field gel electrophoresis (PFGE) analysis

PFGE was done as described in Argueso *et al.* (2008). To quantify the percentage of chromosome II pulsed field gels were stained with SybrGold (Invitrogen), and imaged using a Bio-Rad Gel doc XR+ imaging system. The image was analyzed with image lab 5.0 (Bio-Rad). First, the lanes were detected, followed by detection of the bands. The volumes of all bands were calculated according to the band intensity by Image Lab. Chromosome II volume percentage was calculated from the total of volumes of chromosomes II, X, and XIV.

### Data availability

All data and strains are available; information is found in Supplemental Material. A list of strains is detailed in Table S1 in File S1. A list of primers used to construct the different strains is provided in Table S2 in File S1. The median rates and confidence of intervals are detailed in Table S3 in File S1. Figure S1 in File S1 contains genotyping of the *URA3* locus. Figure S2 in File S1 contains the original images of PFGE analysis.

## Results

**Figure 1:**
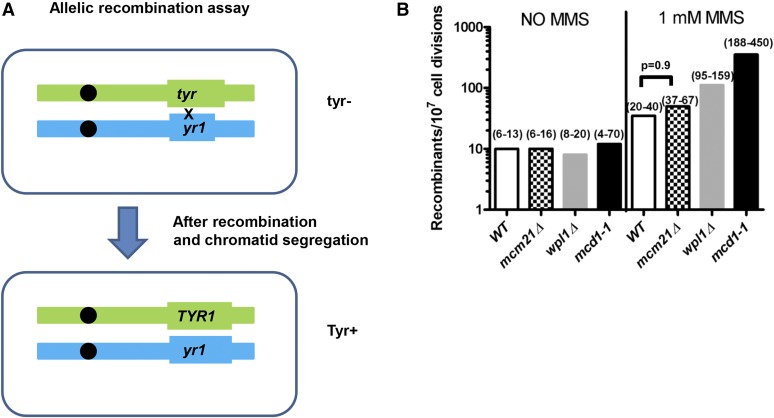
Mcd1 and Wpl1 but not Mcm21 are important to maintain recombination fidelity away from the centromere (A). An assay to measure interhomolog (allelic) recombination as previously described (Covo *et al.* 2010). Each homolog of chromosome II bears a different truncated allele of *TYR1* that is located on the right arm ∼350 kb from the centromere. Since the alleles share a 400 nt overlap, IR can restore the *TYR1* gene. The recombination rate is calculated from the number of tyrosine prototroph colonies among total survivor colonies. For simplicity, only one scenario of recombination that occurs at G1 is illustrated. (B). Cultures of each genotype were grown overnight on solid medium with or without 1 mM MMS, then spread over tyrosine^–^ and complete plates. The bar height represents the median (95% C.I. in parentheses).

We had previously reported that hypomorphic mutants in the cohesin complex increase the frequency of allelic recombination when cells are irradiated at G2 (Covo *et al.* 2010, 2012a). To test the ability of SCC to suppress allelic recombination under different conditions, we measured allelic recombination in cells that were growing under chronic exposure to the DNA damaging agent methyl methanesulfonate (MMS). We also measured the effects of regulators of cohesin on allelic recombination. Both *mcd1-1* and *wpl1Δ* cells showed increased allelic recombination compared to wild type when cells were exposed chronically to MMS (Figure 1). In contrast, as expected, *mcm21*Δ did not show a significant increase in the rate of allelic recombination (this assay measures allelic recombination in a region ∼350 kb away from the centromere).

### Defects in SCC lead to LOH primarily through CL, and only modestly through GC

**Figure 2:**
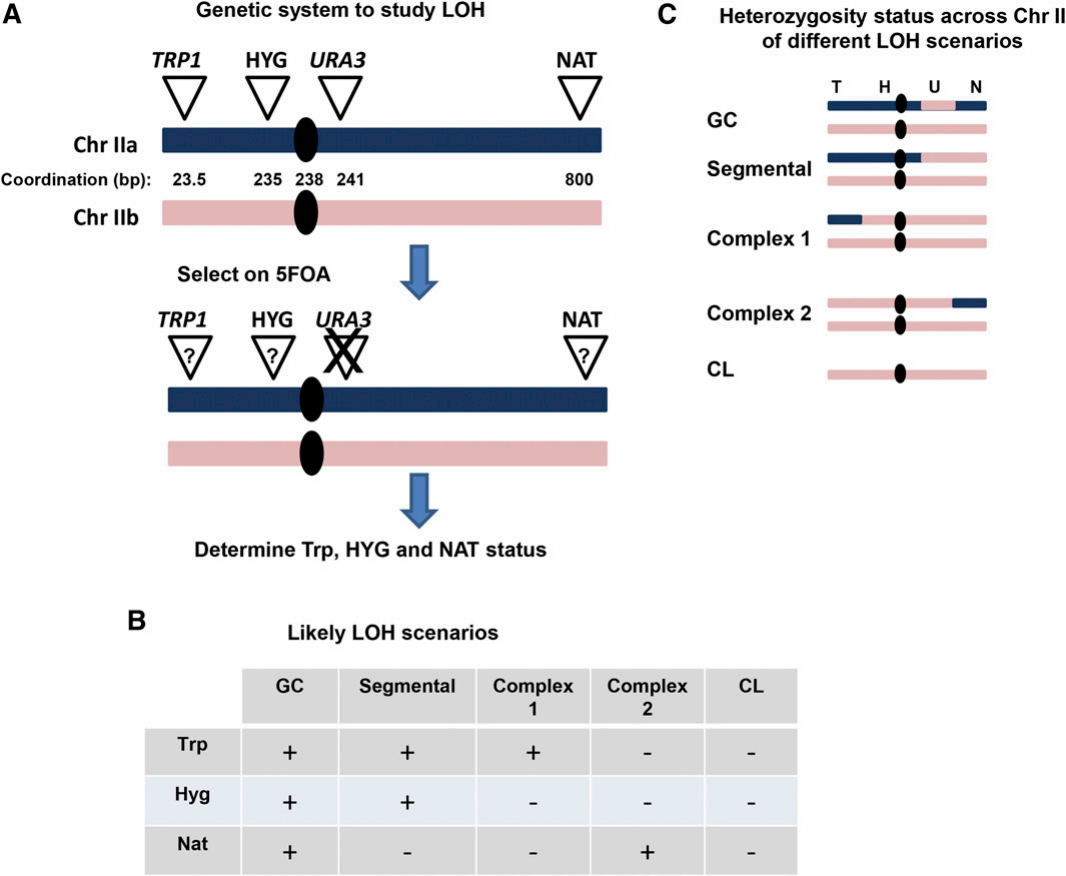
A genetic assay for LOH in diploid yeast cells. (A) In a diploid yeast strain, one homolog of chromosome II was modified by ectopic insertion of four genetic markers: *TRP1*, *HPH* (Hygromycin resistance), *URA3*, and *NAT* (Nourseothricin resistance). The loci of all markers are indicated across the chromosomes (numbers in kilobases). the LOH assay was carried out in two stages. First, cells that lost *URA3* were selected on 5FOA medium; second, the retention of all other markers was determined in the 5FOA-resistant colonies. (B) Four likely scenarios (classes) for LOH according to the phenotype. (C) The expected structure of both chromosome II homologs of 5FOA resistant cells at G1 for each scenario is illustrated. This assay does not reveal the nature of segmental events. Similarly, the exact nature of the complex events cannot be fully understood using the phenotypes of the survivor colonies alone.

While defects in SCC can increase interhomolog recombination, which could be a source of LOH, the extent to which they alter the pattern of recombination is not clear. To address the role of SCC in LOH that might be due to CL or various types of recombination, we used a recently developed diploid LOH assay where one homolog of chromosome II contains four ectopic genetic markers: two are telomeric (*TRP1* gene and resistance to Nourseothricin, NAT^R^), and two are close to the centromere (*URA3* and hygB^R^, resistance to hygromycin) (Covo *et al.* 2014a). The annotation of these markers is detailed in Figure 2 (for further details see Covo *et al.* 2014a). LOH events at the *URA3* locus are selected by resistance to 5FOA (5FOA^R^). Since this assay selects primarily LOH around the centromere, the role of centromeric SCC in LOH prevention can be addressed directly. The 5FOA^R^ colonies are then examined for retention of the other markers in order to deduce the mechanism of LOH. Several possible scenarios for LOH were considered based on marker retentions. Loss of all markers (*TRP1*, HYG^R^, and NAT^R^) is most probably due to CL. Retention of all markers but the *URA3* gene is most probably due to GC (see below for evidence supporting GC scenario over a mutation in the *URA3* gene and the dependence of GC on *RAD51*). Loss of the markers from the right arm (*URA*3 and NAT^R^) is probably due to one of several recombination events: reciprocal cross-over, half cross-over, or break-induced replication. This assay cannot distinguish between these different scenarios; therefore, they were classified as segmental LOH. One type of event which was frequently observed was more difficult to explain; retention of one telomeric marker (NAT^R^ or *TRP1*) and loss of the others. These events were classified as complex. The possible scenarios leading to these complex events are discussed below.

**Figure 3:**
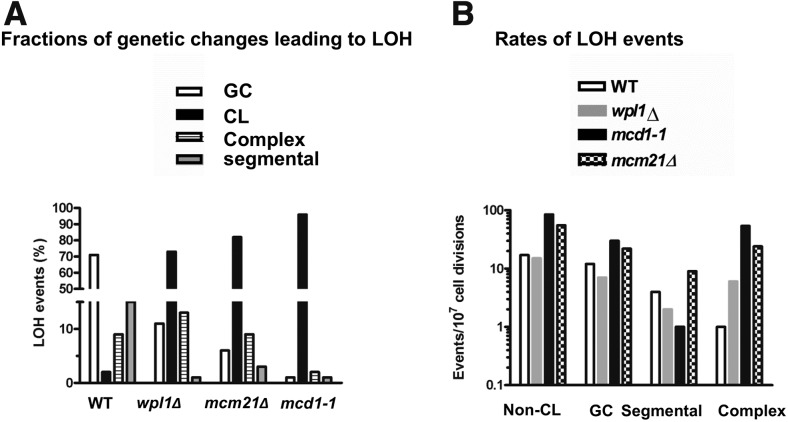
SCC-defective strains show high frequencies of CL and complex events (A). The pattern of LOH was determined using the definition in Figure 2C for several 100 independent (from different cultures) 5FOA-resistant colonies for each genotype; results are presented as percentage of total colonies (WT, 395 total colonies; *wpl1Δ*, 450 colonies; *mcm21Δ*, 200 colonies; and *mcd1-1*, 390 colonies). (B) The median rate for each LOH scenario for each genotype was determined from at least six independent cultures (for 95% C.I., see Table S3 in File S1). 5FOA-resistant colonies were picked to YPDA plates and incubated (arranged in 100–200 colonies a plate); the YPDA plates were then replica-plated to HYG, URA–,TRP– and NAT plates. Since CL events were so frequent in *mcd1-1* cells, the lawn of 5FOA-resistant colonies was replica-plated to NAT or TRP^–^ plates, then 5FOA^+^ NAT^+^ or 5FOA+ TRP+ colonies were transferred to YPDA plates. Colonies were further analyzed by replica-plating as described above. The medians of non-CL rates were calculated by subtracting CL rates from total LOH rates (both rates are presented in Table S3 in File S1).

The nature of genetic changes in several 100 spontaneously arising 5FOA^R^ independent colonies (each colony was from a different culture) is presented in Figure 3A. In WT cells, the most frequent source of LOH (70% of all events) was GC of the *URA3* locus. Modest defects in SCC due to *wpl1*Δ (SCC regulator) and *mcm21Δ* (centromere specific SCC factor) mutations resulted in a very different pattern, where most LOH events were due to CL, although ∼10–20% can be explained by recombination or other mechanisms (segmental LOH and complex events) (Figure 3A). Finally, the LOH in the temperature-sensitive *mcd1-1* cells growing under semipermissive conditions (30°) was primarily due to CL (96%).

Since the overall rate of LOH in *mcd1-1* cells was more than two orders of magnitude higher than in WT (Table S3 in File S1), the analysis of events in independent isolates may result in an underestimate of events other than the predominant CL. Therefore, we determined the rates of the different types of LOH for each SCC mutant using the fluctuation test, calculating the median rate and 95% C.I. The 5FOA^R^ colonies from at least six cell cultures were examined for associated genetic changes. As expected, total LOH was 10- to 200-fold higher in all SCC-defective cells (see Table S3 in File S1 for 95% C.I.), mainly due to CL. The median CL differed considerably between WT, *wpl1*Δ and *mcd1-1* cells: 5, 107 and 5531 events per 10^7^ cell divisions, respectively. Except for complex events (as described below), the rate of total events attributed to recombination in SCC mutants was of the same order of magnitude as the WT strain. Specifically, the GC and segmental LOH rates were comparable across the WT and all SCC-defective strains, or increased up to threefold in the latter. For example, GC rates for WT and *mcd1-1* were 12 and 30 events per 10^7^ cell divisions, respectively (Figure 3B).

To confirm that loss of *URA3* (TRP+/HYG+/NAT+) event is indeed due to GC, we measured the rates of 5FOA resistance in WT and *mcd1-1* haploid strains. The WT haploid rate was 0.8 (0.3–3) × 10^−7^, and the *mcd1*-1 rate was 5 (2–9) × 10^−7^. In both WT and mutants, the haploid rate was at least one order of magnitude lower than the diploid one; therefore, the contribution of mutations in the *URA3* gene to the phenotype of ura–, TRP+ HYG+ NAT+ is neglected. The 5FOA resistance rate was calculated in haploid and diploid *rad51* null strains; unlike WT strains the rate of the ura–, TRP+ HYG+ NAT+ phenotype was similar between the ploidies 13 *vs.* 15 events / 10^7^ (Table S3 in File S1). Moreover, the genotype at the *URA3* locus of 10 ura–, TRP+ HYG+ NAT+ from WT strains and from *rad51*Δ/ *rad51*Δ was determined by PCR. While in nine out of 10 colonies in WT the full-length *URA3* could not be identified, it was identified in nine out of 10 isolates from the *rad51* deficient strain (Figure S1 in File S1). The explanation is that, in WT, 5FOA resistance is due to loss of the entire *URA3* gene by recombination, and in *rad51* null strains, it is through mutations.

In attempts to explain the lower than expected GC rates in *mcd1-1*, we hypothesized that the *URA3* ectopic insertion created a gap in the homology between the two chromosomes; this gap could interfere with recombination. Therefore, another *mcd1-1* diploid strain was constructed, in which a *ura3* nonsense allele was inserted opposite the *URA3* insertion on chromosome IIb. The total LOH rate for this *ura3nonesense/URA3* strain was of the same order of magnitude as the *ura3∆/URA3* strain. The median rate of total LOH was ∼4 × 10^−4^ in *ura3nonsense/URA3* *vs.* median rate of LOH of ∼6 × 10^−4^ in *ura3∆/URA3* (Table S3 in File S1). There was also no major difference in the rates of CL. There was an expected threefold increase in GC rate between the *ura3nonsense/URA3* and *ura3∆/URA3* strain (Table S3 in File S1).

The low rates of recombination-mediated LOH could stem from the centromere proximity of the *URA3* gene. Therefore, we generated diploid strains where the *URA3* gene was inserted next to the NAT cassette at position 795,000 on the right arm. The rate of LOH in the WT diploid strain with this modification was indeed much higher than the centromeric *UAR3* 8 (6–11) × 10^−5^ telomeric marker *vs.* 20 (11–43) × 10^−7^ centromeric marker (Table S3 in File S1). In WT diploids, almost all of the LOH events were segmental (*i.e.*, TRP+ HYG+ ura– nat–), as expected from cross-over or break-induced replication. Unlike WT cells, in *mcd1-1* mutants positioning of the *URA3* cassette next to the telomeres had much less effect on total LOH rates [50 (30–90) × 10^−5^ telomeric marker *vs.* 55 (33–77) × 10^−5^ centromeric marker, Table S3 in File S1]. Even when using this strain, most of LOH events were CL 40 (30–60) × 10^−5^ (Table S3 in File S1). The rate of segmental LOH was comparable to WT cells 12 (10–40) × 10^−5^.

### Defects in SCC lead to high rate of complex events

**Figure 4:**
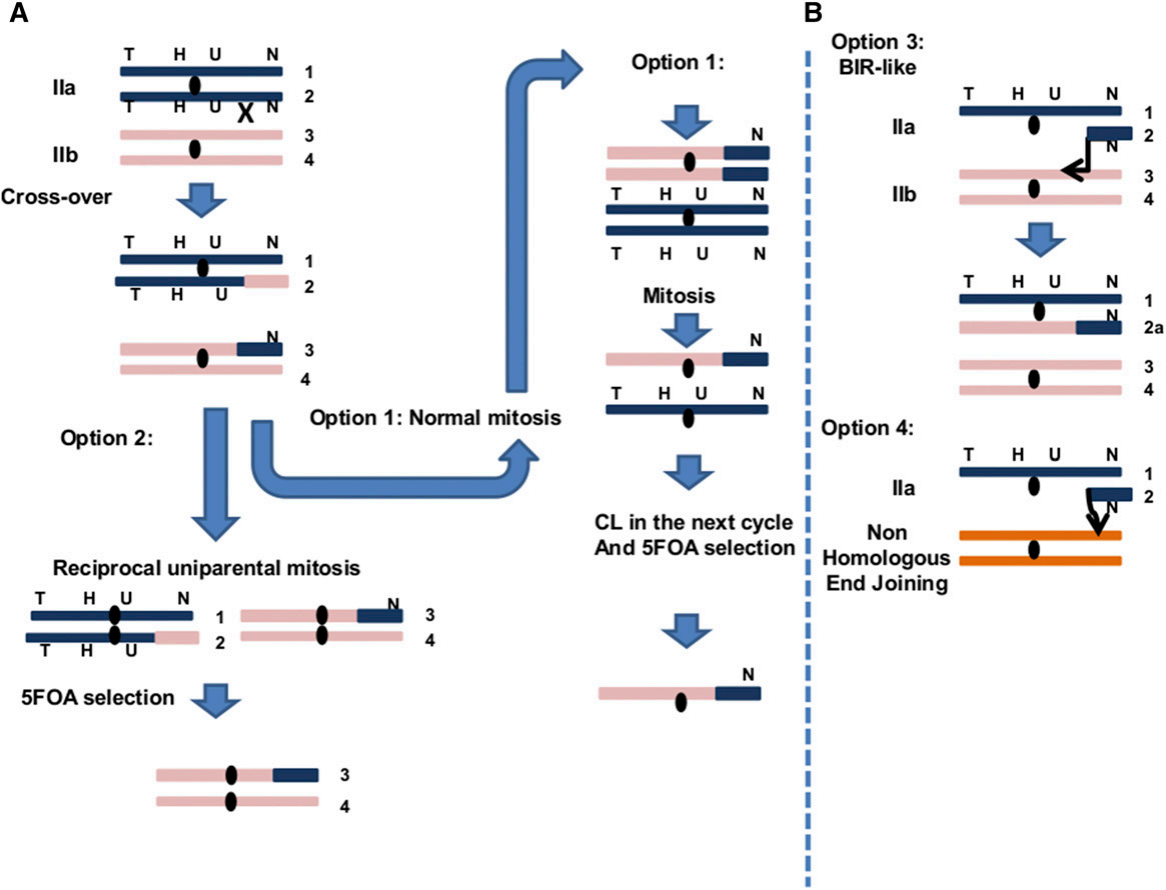
Possible scenarios for the formation of complex LOH events. Four possible scenarios were considered to explain the LOH complex event. (A) First, a crossover between the centromere and the telomere occurs, then two alternative options were considered. Option 1: normal mitosis and CL of chromosome IIa in one of the next cell divisions. This scenario ends up with homozygote locus of the telomeric marker (NAT or *TRP1*). Option 2: instead of a normal mitosis right after crossover, a reciprocal uniparental mitosis occurred in which homologous chromosomes rather than sister chromatids were segregated to the different poles. This scenario ends up with heterozygote locus of the telomeric marker (NAT or *TRP1*). (B) Double strand break in chromosome IIa, followed by loss of the centromeric part and rescue of the telomeric part. Rescue can occur in two manners: invasion of a telomeric fragment of chromosome IIa to chromosome IIb, and establishing a replication fork in a BIR-like reaction, option 3. Alternatively, the telomeric part can be captured ectopically by nonhomologous end joining (option 4). In both options 3 and 4 the telomeric locus is in a heterozygous state.

**Figure 5:**
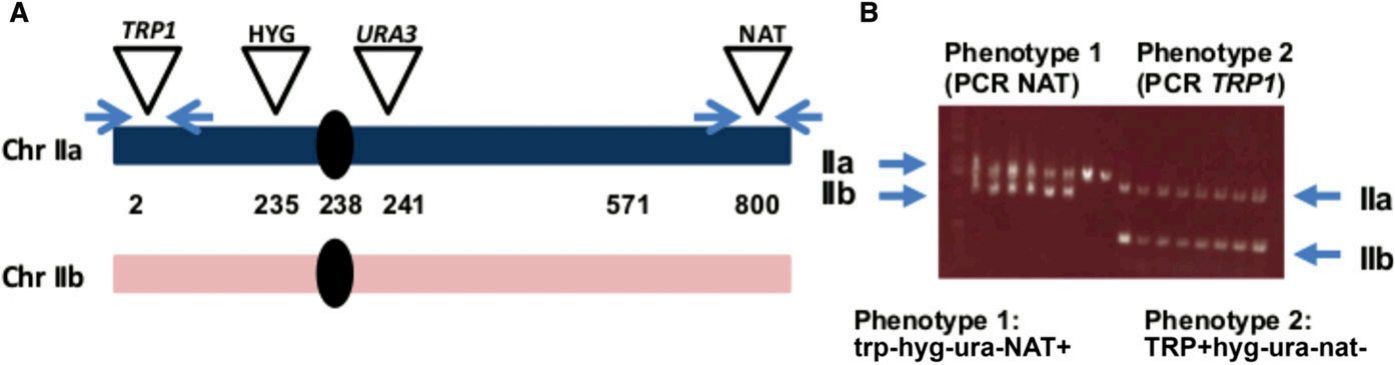
Significant portion of all complex events in *mcd1-1* mutants show heterozygosity for the telomeric marker. Schematic representation of the LOH system, which is similar to Figure 2A. Inverted arrows indicate the primers that were used to amplify the NAT or *TRP1* locus in order to check heterozygosity. (B). For each phenotype (1 or 2), one PCR reaction was done at the locus of the retained marker (NAT OR *TRP1*) using one of the primer sets indicated in (A). A sample of such PCR reactions is shown. The presence of two PCR products is in agreement with either a BIR/NHEJ scenario or reciprocal uniparental mitosis (see text and Figure 4).

**Figure 6:**
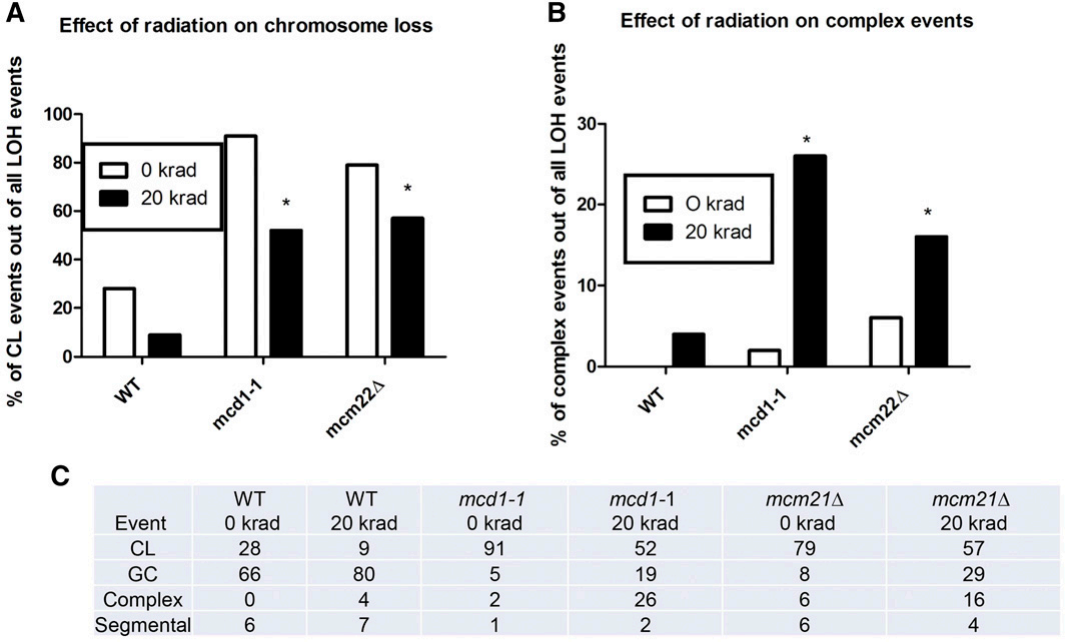
(A)–(C) Ionizing radiation causes increase in the proportion of complex events (B) over CL events (A) in *mcd1-1* cells. G2 arrested cultures with the indicated genotypes were plated using a pronging device to YPDA plates and either irradiated (20 krad) or not. After 3 d the plates were replica-plated into FOA plates, and resistant colonies from mini-cultures were tested for retention of NAT, HYG, and TRP markers. A limited number of colonies (50) was analyzed for the nonirradiated WT culture because there was not much difference from the spontaneous events presented in Figure 3. For SCC-defective cultures, whether or not they were irradiated, 150 colonies were analyzed.

We further analyzed the LOH complex events in strains containing centromeric *URA3* marker (trp– hyg– ura– NAT+ or TRP+ hyg– ura– nat–). Surprisingly, complex events were much more frequent in SCC compromised cells including *mcd1-1* with rates that were 6- to 54-fold greater than in WT (Figure 3B and Table S3 in File S1). High rates of complex events were observed in *mcm21* deficient cells: one *vs.* 24 events/per 10^7^ cell divisions for WT *vs.* *mcm21*Δ. The easiest way to explain these events is by cross-over between *URA3* (centromere) to the NAT locus (telomere) in one cell division, followed by loss of chromosome IIa in another cell division (Figure 4, option 1). However, there are two pieces of evidence that argue against this scenario. First, the result of multiplying the rates of segmental recombination between the centromere and the telomere and CL, as expected from events that occur independently, gives a median value of ∼7 × 10^−8^, while the median of the observed rates of the complex events is ∼6 × 10^−6^. The differences between these values is statistically significant using a binomial test (*P* < < 0.01). The rate of recombination between the centromere and the telomere in *mcd1-1* diploids was based on the rate of TRP+ HYG+ ura– nat– colonies when the *URA3* gene was placed next to the telomere (Table S3 in File S1). The second and stronger argument is the fact that we could observe heterozygosity at the telomeric segment of the complex events; as seen below; *i.e.*, either heterozygous for NAT in cells that are trp– hyg– ura– NAT+ or heterozygous for *TRP1* in cells that are TRP+ hyg– ura– nat–. Using the PCR analysis as described in Figure 5, heterozygosity for the NAT locus was observed in 17/27 independent events and 7/7 for the *TRP1* locus examined in the *mcd1-1* mutant. An alternative mechanism to explain the complex events is reciprocal uniparental segregation of chromatids preceded by cross-over between the centromere and the telomere (Figure 4A, option 2) (Andersen and Petes 2012). However, this scenario is unlikely due to the high rate of the complex events. Alternatively, there are two scenarios that stem from a common starting point: a break that occurred in chromosome IIa, after which the telomeric side survived and the rest of the chromosome is lost (Figure 4, option 3, 4). To address this possibility, the status of NAT, HYG, and TRP markers of several 100 independent 5FOA^R^ colonies in WT and SCC-deficient diploid cells induced by ionizing radiation was determined. Because selection on 5FOA does not allow rate determination of (IR) ionizing radiation-induced LOH events, only the changes in the distribution between the different scenarios are presented. Since effects of SCC on recombination are limited to cells that already duplicated their chromosomes, IR-induced changes were examined in G2-arrested cells. To ensure that the arrest itself did not change the LOH pattern, independent isolates from arrested but unirradiated cultures were also examined. For unirradiated *mcd1-1* cells, CL accounted for ∼90% of the 5FOA^R^ colonies and complex events only ∼2%. Following irradiation, the CL events dropped to 52% (Figure 6A) and the percentage of complex events increased to 26% (Figure 6B). A similar, though less dramatic trend was observed for *mcm21Δ* cells. The CL events were reduced from 80 to 57% (*P* value 0.0004 corrected for multi-hypothesis testing, Figure 6A), and complex events increased from 6 to 16% (*P* value 0.04 corrected for multi-hypothesis testing, Figure 6B). The impact of IR on complex events was not as clear in the WT cells, since there was a predominance of GC with and without radiation.

### Analysis of complex events in mcd1-1 and rad51Δ cells

**Figure 7:**
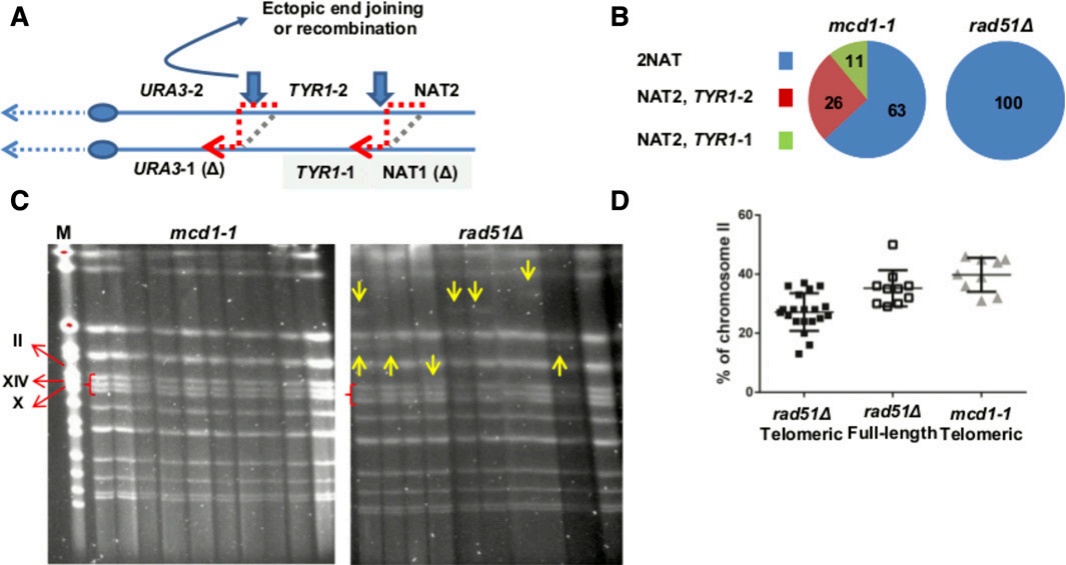
Genotypic analysis of complex events in *mcd1-1* and *rad51*Δ cells. trp– hyg– ura– NAT+ colonies from *mcd1-1* and *rad51Δ* backgrounds were genotyped at the NAT and *TYR1* loci using primers 29 and 30. (A) NAT2, *TYR1*-2 and *URA3*-2 correspond to the alleles shown on chromosome IIa in Figure 2 and Figure 4. Vertical arrows indicate potential break sites. The gray box highlights recessive alleles that are lost during either a half crossover (dashed line) or BIR reaction (red arrowed dashed line). Curved arrow symbolizes an ectopic NHEJ reaction. (B) Distribution of genotypes regrading to NAT and *TYR1* loci in *mcd1-1* and *rad51*Δ . (C) An example of pulsed field gels of trp– hyg– ura– NAT+ colonies from *mcd1-1* and *rad51Δ* backgrounds. Arrows indicate chromosome aberrations; red braces highlight chromosomes II, XIV, and X. All pulsed field gels, including the one done for TRP+ URA+ HYG+ NAT+ colonies from *rad51Δ* background, are presented in Figure S2 in File S1. (D) The percentage of chromosome II band intensity out of the sum of intensities of chromosomes II, XIV, and X as observed in pulsed field gels (Figure S2 in File S1). This percentage was calculated for 20 *rad51Δ* trp– hyg– ura– NAT+ (telomeric) colonies; 10 *rad51Δ* TRP+ URA+ HYG+ NAT+ (full length) colonies and 10 *mcd1-1* trp– hyg– ura– NAT+ (telomeric) colonies (see *Materials and Methods*).

We further analyzed the formation of the complex events in *mcd1-1* and *rad51*null strains. The rate of former events was about five times higher than the latter (54 *vs.* 11 events/10^7^ Table S3 in File S1), but, in both cases much higher than WT. The rationale of the experiments presented below is to examine the mechanism of formation of the complex events in *mcd1-1* and *rad51* null mutants. Our hypothesis is that, while in the former, complex events are due to recombination, in the latter they are due to ectopic end joining. In both mutants, the *HYG* and *URA3* alleles were lost, indicating that the phenotype does not stem from point mutations in these genes. Genotyping using molecular markers along the right arm of chromosome II revealed interesting differences between colonies with the phenotype of trp– hyg– ura– NAT+ of *mcd1-1* and *rad51*null strains (Figure 5 and Figure 7). First, while in *mcd1-1*, 17/27 events were heterozygous, at the NAT locus all 22 *rad51* null colonies were heterozygous (Figure 7, A and B, Fisher exact test *P* = 0.0011). In our system, there are two *TYR1* truncated alleles (Figure 1) positioned ∼230 kb from the right telomere (NAT side). We genotyped the *TYR1* locus in trp– hyg– ura– NAT+ of *mcd1-1* and *rad51*null strains. In *rad51* null, all events showed two PCR alleles. In *mcd1-1* there were 3/27 events of NAT-2 allele and *TYR1*-1 allele, suggesting a half crossover event that occurred between the NAT and *TYR1* loci. There were 7/27 events of NAT-2 allele and the *TYR1*-2 allele, suggesting half crossover between the *TYR1* and the *URA3* loci (Figure 7). Surprisingly, we were not able to observe a case in which the NAT locus was heterozygous, and the *TYR1* locus was homozygous (Figure 7, A and B). These results allow us to hypothesize that, in *mcd1-1* mutants, an invasion of a broken telomeric segment containing the NAT cassette to the homologous chromosome (IIb) occurs. In some cases this invasion progresses by break-induced recombination across the centromere to the left arm telomere. In others, the BIR is resolved to half crossover (Figure 4 and Figure 7A). As suggested above, chromosome IIa is lost.

BIR, or any other recombination mediated repair, is almost always not possible in *rad51* null strains. In these strains, nonhomologous end joining can generate trp– hyg– ura– NAT+ colonies (Figure 7A) by ectopically fusing the right telomeric segment of chromosome II elsewhere in the genome while the chromosome itself is lost. We analyzed trp– hyg– ura– NAT+ colonies heterozygous at the NAT and *TYR1* loci. The size of a chromosome fragment that contains both the NAT and *TYR1* loci is ∼230 kb, and, therefore, the result of ectopic translocation of this fragment should be observed by PFGE. We expected that in *mcd1-1* background these colonies will not show any structural variation because the homologous chromosome is used as a template. In addition, in the *mcd1-1*case, invasion of the telomeric fragment to the homologous chromosome using it as a template will restore the centromeric fragment of the chromosome. We separated the chromosomes of isolates using PFGE and found that, unlike *mcd1-1* isolates, many structural variations can be observed in the *rad51* null (Figure 7C). Fewer chromosomal aberrations are seen in *rad51* null colonies with the phenotype of TRP+ HYG+ URA+ NAT+ (Figure S2 in File S1). At this point we do not know what are the structural variations that are directly associated with ectopic translocation of the telomeric fragment of chromosome II. To estimate the copy number of chromosome II, we calculated the intensity of the band corresponding to chromosome II. To adjust lane to lane differences, the chromosome II intensity was divided by the sum of intensities of the band of chromosomes II, XIV, and X. We observed a reduction in the intensity of chromosome II band in *rad51* null colonies with the phenotype of trp– hyg– ura– NAT+ in comparison with *rad51* null colonies with the phenotype of TRP+ HYG+ URA+ NAT+ or *mcd1-1* colonies with the phenotype of trp– ura– hyg– NAT+ (Figure 7D, *P* = 0.03 and 0.001, respectively).

#### POL32 dependent CL in mcd1-1:

**Figure 8:**
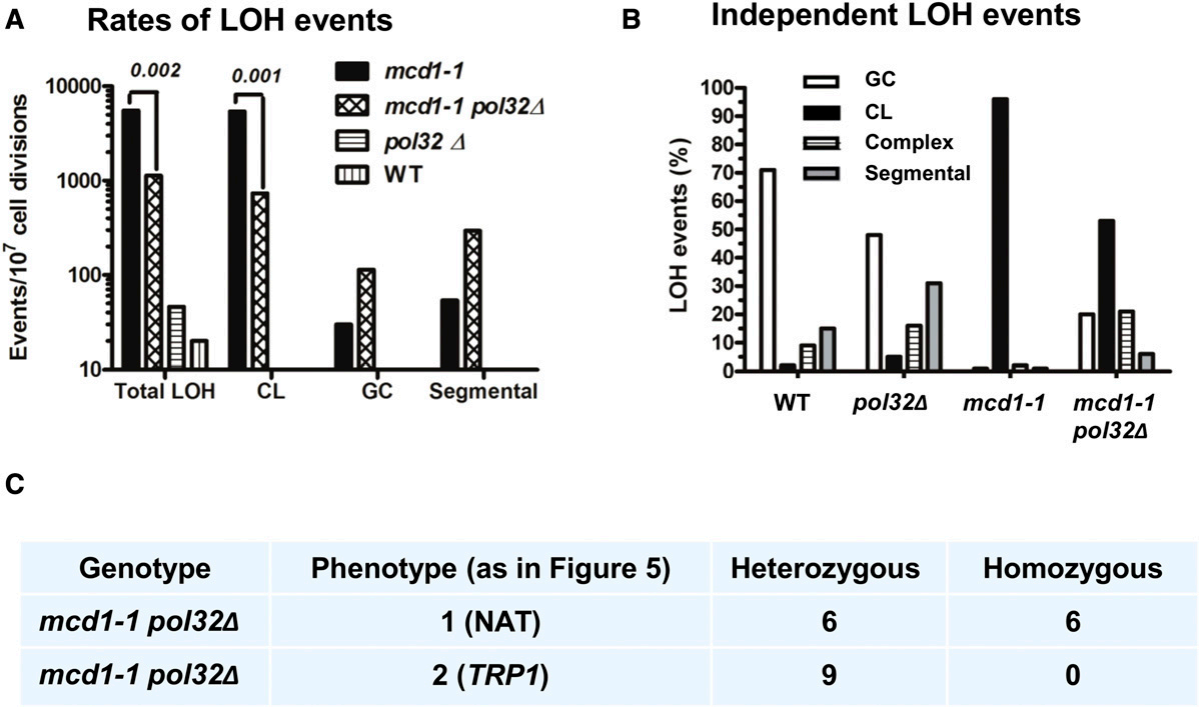
(A)–(C) Pol32 is not essential for the formation of complex events in *mcd1-1* mutants but affects CL rates. (A) The rate for total LOH, CL, GC, and complex events was determined for the *mcd1-1 pol32Δ* double mutant (same as Figure 3). Star “*” indicates *P*-values < 0.05 in a *t*-test. There was no significant difference in the rate of complex events between *mcd1-1* single mutant and *mcd1-1 pol32Δ* double mutant (*t*-test, *P*-values = 0.2). (B) The LOH pattern that was obtained from 308 and 268 independent 5FOA^R^ colonies for *mcd1-1 pol32Δ* and *pol32Δ*, respectively, is presented (results for WT and *mcd1-1* are taken from Figure 3A). White bars, GC; Black bars, CL; Gray bars segmental recombination; Striped bars, complex events.

Our data suggest that complex events could be formed by BIR. Based on results with a nuclease-induced DSB, BIR is largely dependent upon *POL32* (Jain *et al.* 2009; Ruiz *et al.* 2009). In order to examine if Pol32-dependent BIR plays a role here, *POL32* was inactivated in the *mcd1-1* background. We determined the spontaneous LOH pattern in a *mcd1-1 pol32Δ* double mutant. There was no reduction in the rate of complex events based on genetic analysis, in fact, there is an increase in the rate (more experiments are needed to determine if this difference is significant, Figure 8 and Table S3 in File S1). We could still observe comparable events of heterozygosity of the telomere segments among the complex events (*pol32Δ mcd1-1* double mutant, Figure 8). In contrast, there was an ∼5- to 10-fold decrease in total LOH and CL rates in the *mcd1-1 pol32Δ* cells as compared to the single *mcd1-1* mutant (*P* = 0.001; Figure 8). Moreover, the LOH pattern of the double mutant is much closer to *mcm21Δ* or *wpl1Δ*-deficient cells than *mcd1-1* with a far lower proportion of CL events in single isolates from independent *mcd1-1 pol32Δ* cultures (cf. Figure 8 right side with Figure 3A).

Since deletion of *POL32* could have an effect on CL that is independent of defects in cohesin, LOH was measured in a *pol32Δ* single mutant. Unlike the *mcd1-1 pol32Δ* double mutant, the total LOH rate in the single *pol32Δ* mutant cells was higher than the WT rate (46 (35–64) *vs.* 20 (11–43) events per 10^7^ cell divisions, Table S3 in File S1). Thus, the reduction in total LOH for the *mcd1-1 pol32Δ* double mutant is not attributable to an independent effect of *pol32Δ*. The rate of CL in *pol32Δ* was very similar to WT [3 (2–4) *vs.* 5 (1–8) events per 10^7^ cell divisions, Table S3 in File S1]. Since the fluctuation tests are not ideal for relatively small changes, we tried to determine the differences in LOH among the different mutants by examination of independent events. Examining the LOH patterns in the independent 5FOA^R^ survivors that were *pol32Δ* or WT (Figure 8B), there was no significant difference in the proportion of CL events between the two strains. Therefore, the reduction in the CL rate in the *mcd1-1 pol32Δ* is not due to an independent effect of *pol32*Δ. The proportion of segmental recombination events was significantly higher in *pol32Δ* cells compared to WT −30% *vs.* 15% (*P* = 0.0004, *χ^2^* test corrected for multiple hypothesis testing; Figure 8). On the other hand, GC events were significantly lower −48% in *pol32Δ* *vs.* 71% in WT (*P* = 0.0004, *χ*^2^ test corrected for multiple hypothesis testing). As described below, *pol32Δ* exhibits higher proportion of complex events. Thus, while *POL32* influenced recombination independently, its dramatic effect on CL was only observed in the *mcd1-1* background. Possible explanations for these results are discussed below. The difference shown in the distribution of independent LOH events between WT and *pol32* Δ (shifted toward segmental events) was not reflected in the rate measurements (Table S3 in File S1); this is probably due to a minor effect that is not observed in fluctuation tests. However, there is a difference in the rate of complex events between WT and *pol32*Δ [1 (<1–2) *vs.* 14 (5–40), Table S3 in File S1]. At this stage we do not know the reason for this difference.

## Discussion

### Defects in SCC cause LOH mainly through CL under normal growth conditions

A prominent impact of SCC is the suppression of whole chromosome aneuploidy under normal growth conditions (Figure 3 and Table S3 in File S1). The low contribution of other events like GC or segmental recombination (cross-over or BIR) to LOH could be due to relatively few DSBs that might initiate recombination under those conditions. Since we use hypomorphic SCC mutants, the few breaks that might form could be repaired by the sister chromatids preventing the appearance of recombination-associated LOH. It seems that once cells are exposed to DNA damage, the role of SCC in preventing allelic recombination becomes a little more significant (Figure 1). Nevertheless, even when cells were exposed to DNA damage, the effect of SCC deficiency on recombination was relatively mild (Figure 1 and see Covo *et al.* 2010, 2012b; Tittel-Elmer *et al.* 2012). In contrast, the effect of SCC deficiency on CL was very high (up to 1000-fold increase in rate, Figure 3 and Table S3 in File S1). We recently determined the effect of *mcd1-1* mutation chromosome gain; like CL, the rates of chromosome gain were much higher in *mcd1-1* mutant compared to WT (Covo *et al.* 2014b). Moreover, DNA damage caused increase in the rate of chromosome gain in cohesin mutant much more than its effect on allelic recombination, as reported previously and here (Covo *et al.* 2010, 2012a, 2014b). We could not observe any dramatic difference between WT and *mcd1-1* mutant regarding the rate of segmental recombination for the telomeric marker despite the fact that there are many more recombinogenic breaks in this case in comparison with the centromeric marker (Table S3 in File S1). In conclusion, the effect of sister chromatid cohesion on homologous recombination is limited.

### Unexpected complex LOH events are revealed in SCC mutants

Using our LOH assay (Figure 2), we identified a new role for cohesin in suppressing complex LOH. At least part of the complex events, the ones that show heterozygosity at the NAT^R^ or *TRP1* loci (Figure 5 and Figure 7) are not due to trivial cross-over followed by CL. At this stage we do not know how these events are generated. Our preferred model for *mcd1-1* strains is invasion of telomeric segment of chromosome IIa (Figure 2 and Figure 4) to the homologous chromosome IIb followed by a break-induced replication reaction till the telomere of the other arm. Two arguments support this model indirectly. First, the proportion of complex events is increased in response to IR (Figure 6A), indicating involvement of breaks. Second, the events of trp– hyg– ura– NAT+ are different between *rad51* Δ and *mcd1-1* strains, indicating that, in the latter, at least some of the events are recombination-mediated (Figure 7). In addition, while complex events in *rad51* null cells involve structural variations and frequent loss of chromosome II, structural variations are not seen in *mcd1-1* strains (Figure 7, C and D). One interesting point is the passage of the replication machinery through the centromere during a BIR reaction. Proteins, including the cohesion complex, that are enriched around the centromere may interfere with unscheduled DNA replication ((Ng *et al.* 2009; Stephens *et al.* 2011, 2013 and references therein). It is possible that the effect of *mcd1-1* and *mcm21* mutants on the formation of the complex events stems from a weaker binding of the cohesin complex, and, therefore, a more relaxed chromatin environment around the centromeres.

The complex events are independent of *POL32*, and, therefore, are probably not due to canonical BIR. It is noted that, even though BIR is generally considered *POL32* dependent, the degree of dependence varies and BIR can also occur in its absence (Lydeard *et al.* 2007; Deem *et al.* 2008; Ruiz *et al.* 2009; Ho *et al.* 2010). Surprisingly, unlike the complex events, the CL rates in the *mcd1-1 pol32* Δ double mutant were lower than in the *mcd1-1* single mutant (Figure 8). The effect of a mutation in *POL32* on CL events in the *mcd1-1* mutant is intriguing, especially since deletion of *POL32* in the WT background did not reduce CL (Figure 8 and Table S3 in File S1). The results may indicate that monosomy for chromosome II survives less well in a *mcd1-1 pol32Δ* double mutant compared to a *mcd1-1*single mutant. Alternatively, some defects in DNA replication in the absence of *POL32* may slightly compensate for the deficiency of *mcd1-1* in chromosome transmission.

Currently we are analyzing the formation of the complex events in several single and double mutants including sister chromatid cohesion and nonhomologous end joining. We are also using genomic approaches to understand the genotype of the complex events in high resolution. We expect that these measures will allow us in the future to understand better the mechanism of formation of the complex events. It is possible that, due to their rarity and complexity, these complex events were overlooked in previous studies. For example, the alternative sliding clamp loader Elg1 genetically and functionally interacts with the SSC machinery. *elg1*Δ cells show high rates of cross-over, although based on the genetic assay that was used, some of the events could be similar to ones observed here (Ben-Aroya *et al.* 2003).

LOH events are interesting because they increase genome plasticity, and thus allow combination of different alleles on the same chromosome. For example, segmental or complex LOH allows loss of an allele that confers sensitivity to a drug (*URA3* and 5FOA) while at the same time maintaining an allele for drug resistance (NAT). LOH is expected to create a combination of new phenotypes that are very important in cancer, but also in fungal human and plant pathogens like *Candida albicans* and *Phytophthora caprisi* (Forche *et al.* 2008; Lamour *et al.* 2012).

In summary, we show that defects in SCC primarily cause LOH via CL. Surprisingly, defects in SCC cause greater increases in complex LOH events than localized, short-track LOH events. The finding that both whole CL and complex LOH events affect many genes may explain why defects in SCC are strongly associated with cancer (Solomon *et al.* 2011).

## Supplementary Material

Supplemental material is available online at www.g3journal.org/lookup/suppl/doi:10.1534/g3.117.300091/-/DC1.
