# Supplementary material for: Increased LOH Due to Defective Sister Chromatid Cohesion Is Due Primarily to Chromosomal Aneuploidy and Not Recombination
Source: G3 (Bethesda). 2017 Oct 3;7(10):3305–15. doi: 10.1534/g3.117.300091 (PMC5633381; doi:10.1534/g3.117.300091)
Supplement: Supplementary file 1 [file 3305FileS1.pdf]

Increased LOH due to defective sister chromatid cohesion is due primarily to chromosomal aneuploidy and not recombination

Dror Sagi, Evgeniya Marcos-Hadad , Vinay K. Bari Michael A. Resnick and Shay Covo

Supplementary information

This file includes

Table S1 – strains

Table S2 – primers

Table S3 – LOH rates

Figure S1- PCR of the URA3 locus of 5FOA resistant WT and rad51 null colonies

Figure S2 – Pulsed field gel electrophoresis of complex events

Table S1: Strains used in this study

Strain	Used for	Genotype
CS 1061	LOH/TYR assay haploid	*MATa*, *ade5-1, his7-2, leu2-3 112, lys2 A14, trp1Δ, ura3Δ, met6Δ, tyr1 300-1359*
CS 1219	LOH/TYR assay haploid	*MATα*, *ade5-1 his7-2 leu2-3,112, trp1Δ ura3Δ met2Δ tyr1 1-700 TRP1_ ec,URA3_ec, hygB_ ec, NAT^R _ec*
CS 1120	LOH/TYR assay haploid	As 1061 but *mcd1-1*

CS 1145	LOH/TYR assay haploid	As 1061 but *wpl1::G418*
CS 1242	LOH/TYR assay haploid	As 1061 but *mcm21::G418*
CS 1256	LOH assay haploid	As 1061 but *pol32::G418*
CS 1257	LOH assay haploid	As 1061 but *mcd1-1 pol32::G418*
CS1234	LOH assay haploid	As 1219 but *mcd1-1*
CS1294	LOH assay haploid	As 1120 but *ura3nonesense _ec* (also *mcd1-1)*
CS1221	LOH assay haploid	As 1219 but *wpl1::G418*
CS1245	LOH assay haploid	As 1219 but *mcm21::G418*
CS1261	LOH assay haploid	As 1219 but *pol32::G418*
CS1259	LOH assay haploid	As 1219 but *mcd1-1 pol32::G418*
FCG69	LOH assay haploid	As 1219 but telomeric *URA3*
FCG120	LOH assay haploid	As 1234 (*mcd1-1*) but telomeric *URA3*
FCG222	LOH assay haploid	As 1219 but *rad51::G418*
FCG223	LOH assay haploid	As 1061 but *rad51::G418*
CS2324	LOH/TYR assay diploid	Cross 1061X1219

	(WT)	
CS2338	LOH/TYR assay diploid (*mcd1-1*)	Cross 1120X1234
CS2430	LOH assay diploid (*mcd1-1 ura3nonsense/URA3*)	Cross 1294X1234
CS2328	LOH/TYR assay diploid (*wpl1*Δ)	Cross 1145X1221
CS2353	LOH/TYR assay diploid (*mcm21Δ*)	Cross 1242X1245
CS2401	LOH assay diploid (*pol32Δ*)	Cross 1256X1261
CS2376	LOH assay diploid (*mcd1-1 pol32Δ*)	Cross 1257X1259
FCG	LOH assay diploid (*rad51Δ*)	Cross FCG222XFCG223
FCG155	LOH assay diploid	Cross FCG120XCS1120 (*URA3* telomeric)
FCG73	LOH assay diploid	Cross FCG69XCS1061(*URA3* telomeric)

Detailed description of the strains used in this study with the relevant genotype, ploidy and the use in the paper. TYR assay refers to Tyrosine recombination assay as described in Figure 1. LOH assay refers to the assay described in Figure 2. *ec* refers to ectopic insertion of the *URA3* or *TRP1* gene as described in Figure 2.

Table S2: Primers used in this study

#	Sequence	Description
1	5' ATGTTTACTTCAGCCCTTTTT	*WPL1* knockout FW
2	5' ACGCTAGAAGGCTCATCAAA	*WPL1* knockout REV
3	5' ACCTGGGCCGTCTTAAATTT	*MCM21* knockout FW
4	5' AGCTTGCCTTGCCATTGTTT	*MCM21* knockout REV
5	5' CACCACGTTAATAGCGATCTG	*RAD51* knockout FW
6	5' AGTAGGGTTGCGAGGTATATG	*RAD51* knockout REV
7	5' CGAATAGGTCTATTTTCCACTACGGT	*POL32* knockout FW
8	5' TGTCAAGCGTTCATGTAAATCAG	*POL32* knockout REV
9	5' TTTTGTGATCGGTGTGGCGC GATCTGTGAAATTTAAACTTTT TTGCGGGTGAAACCGAGATTTCTGAAACCGTACGCTGCAGGTCGACGGATCCCC	Insertion of NAT FW
10	5'TCTTTCACAC CTGTGCAAAA TCCAATGGGA GCAATAAATG TAACATATTT TTTTAACATA CCACGGTAGG-ATCGATGAATTCGAGCTCGTTTTCGA	Insertion of NAT REV
11	5' TCAACAAACCATAAATGGTCACTCC	Confirmation of NAT insertion FW
12	5' TGCTCTCGTTCGATGTTTGA	Confirmation of NAT insertion REV
13	5'TTAGTTTTAATGGAAAACAGTTCCTACAAGCGCTATAACATATGAAATATAC ATTAGTCGAATATTAACT-CAGAGCAGATTGTACTGAGAGTGCACC	Insertion of centromere proximal *URA3* FW
14	5'AATTCACGCATCTTTATAGAGTTTATAATGCAAATCTCCGCGCGGGGTAATTTG AA GTCCAATTTTTCCGA-CGCATCTGTGCGGTATTTCACACCGC	Insertion of centromere proximal *URA3* REV
15	5' GGCTTTATGTTATCTTCACCATCA	Confirmation of

		URA3 insertion FW
16	5'GCATTTTTTTCCTACCACATGGC	Confirmation of *URA3* insertion REV
17	5'TTCCGTAAGTAAAACCGTAAACTTGATACGTTTTTATTTTCTTTATT AATAGTAATACTATACACTGTC-CGTACGCTGCAGGTCGACGGATCCCC	Insertion of HYG FW
18	5' 5' TGGGGTTTTAAAGTAGGTCA TATGAGGAAG ACTGGTATGT CTTTTATCTA ACAGTTTTATAAATAGCGTC-ATCGATGAATTCGAGCTCGTTTTCGA	Insertion of HYG REV
19	5' GAAGACAAATTGCAAGTATCCG	Confirmation of HYG insertion FW
20	5' GGAAAGTACAGAACAAGAGCAAA	Confirmation of HYG insertion REV
21	5'GGTGTCACTAACGAAAAATCTAAAGTTTCCTGGAGGACTTTTGTCTGGTTC ATTAATTCGTCCAGTAGACG-CAGAGCAGATTGTACTGAGAGTGCACC	Insertion of *TRP1* FW
22	5'TCTCCAATATTACTGCAGGTTAGTACATTATTTTTTACTCGCAGTTGCTATTTTG GC TAGAGGCTGCACGG-CGCATCTGTGCGGTATTTCACACCGC	Insertion of *TRP1* REV
23	5' GTAATCATCATTGTGGTGTGGAGTGG	Confirmation of *TRP1* insertion FW
24	5' GTACTACTCGGTCCGGACAGTAGGAT	Confirmation of *TRP1* insertion REV
25	5' GATATTTTTAAGGTTTTACCTGATTCAAGAATCCCAACTGATGGAACAGT-CAGAGCAGATTGTACTGAGAGTGCACC	Insertion of telomere proximal *URA3* FW
26	5' TCATCCCCTGATAAAATGTGTAGTGAAATCCCTCTTTGGCGTAACAAGTT-CGCATCTGTGCGGTATTTCACACCGC	Insertion of telomere proximal *URA3* REV
27	5' ACAATGAAAGAGCCCAAAGC	Confirmation of telomeric *URA3* FW
28	5' CAA ATAGCAGCAGTACCTGGA	Confirmation of telomeric *URA3* REV
29	5'AAGGAAAGAGGACAGCATATCCA	*TYR1* genotyping FW
30	5' GCCTAATATTATAGGAAATCAGCA	*TYR1* genotyping REV

Table S3

Table S3 - mutation and LOH rates

Diploids *URA3* centromeric marker

**Events/10^7 Cell Divisions**	**Total**	**GC**	**Segmental**	**CL**	**Complex**
**WT**	20(11-43)	12 (2-28)	2 (*-4)	5 (1-8)	1 (*-2)
rad51Δ	34(28-41)	15 (3-44)[$]	Not detected	7(2-14)	11(7-25)
wpl1Δ	122(76-311)	7(4-10)	2(*-5)	107 (77-311)	6(0.5-10)
mcm21Δ	146 (17-433)	22 (1-49)	9 (*-13)	107(13-363)	24 (1-35) $p=0.0002$
mcd1-1	5534 (3271-7705)	30 (6-50)	1 (*-7)	5531 (3271-7705)	54 (49-131)
pol32Δ	46 (35-64)	28(7-108)	28(3-32)	3 (2-4)	14(5-40)
mcd1-1 pol32Δ	1112 (459-1530)	114 (44-516)	* (*-22)	700 (*-1505)	294 (104-697)

Diploids *URA3* centromeric marker - *ura3nonsense/URA3*

**Events/10^7 Cell Divisions**	**Total**	**GC**	**Segmental**	**CL**	**Complex**
mcd1-1	4000 (3000-7000)	90 (20-100)	ND	3632 (1051-6962)	ND

Haploids *URA3* centromeric marker

**Events/10^7 Cell Divisions**	**Mutation Rate**				
**WT**	0.8 (0.3-3)				
mcd1-1	5 (2-9)				
rad51Δ	13(7-19)				

Diploids *URA3* telomeric marker

Events/10^5 Cell Divisions	Total	GC	Segmental	CL	Complex
**WT**	8 (6-11)	**	8 (6-11)	**	**
mcd1-1	50 (30-90)	**	12 (10-40)	40 (30-60)	**

The median rates and 95% confidence of intervals (in parenthesis) of different LOH scenarios are presented. Six independent cultures or more were used to determine the rates; experiments were done as described in Material and Methods and Results sections. * indicates a rate lower than $5X10^{-8}$ the lowest rate that can be determined under the assay conditions. $ indicates the mutation rate in the *URA3* gene and not GC. While the phenotype of a mutation in the *URA3* and GC is the same, the difference can be identified using molecular genotyping (see Figure S1). Due to its simplicity determining the rate of mutations in the *UAR3* gene in haploids can be done with greater confidence even for lower rates than LOH assay.** indicates a rate lower than $1X10^{-6}$, the lowest rate that can be determined under the experimental conditions, corresponding to the actual number of colonies counted. N.D – the rate was not determined, not to be confused with not-detected were attempts to determine the rate failed because no colonies were grown.

Figure S1

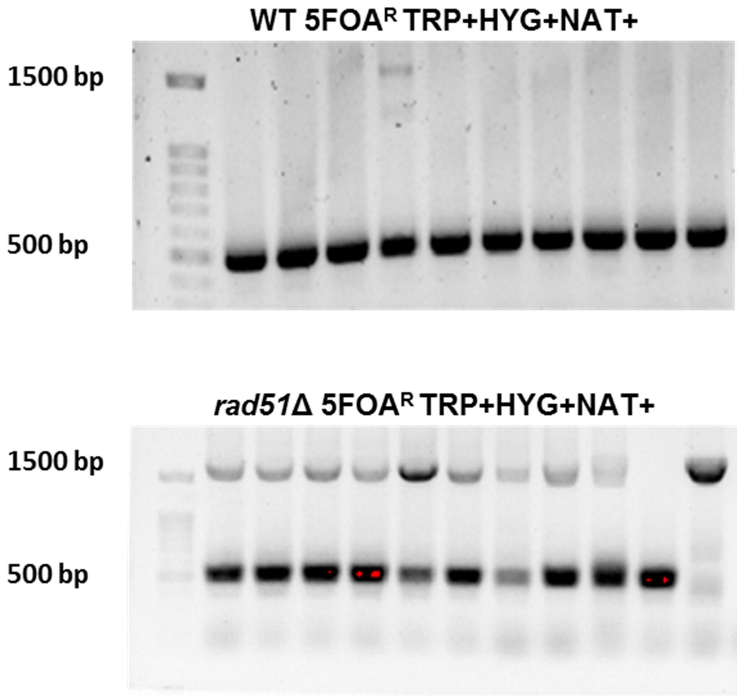

5FOA resistant colonies from WT and *rad51* null strains that maintain TRP+, HYG+ and NAT+ phenotype were examined for the existence of the *URA3* gene using primers 15 and 16 (see Table S2). While in WT 5FOA resistance is due to *URA3* loss because of recombination with the homologous chromosome in *rad51* null strains 5FOA resistance is due to a mutation in the gene.

Figure S2

***rad51*Δ trp- hyg-ura- NAT+**

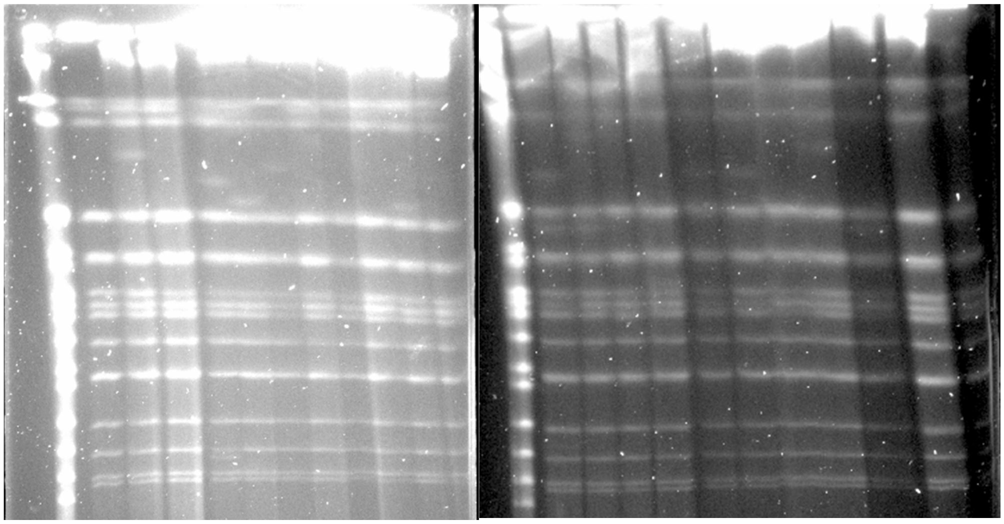

***rad51*Δ TRP+ HYG+URA+ NAT+**

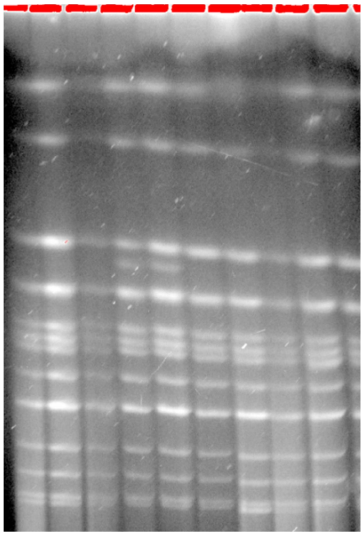

***mcd1-1* trp- hyg-ura- NAT+**

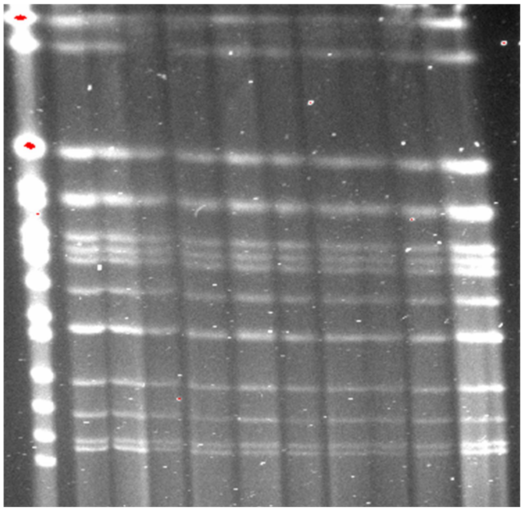

The karyotype of *rad51* null and *mcd1-1* colonies as determined by pulsed field electrophoresis; upper and bottom right gels are presented in Figure 7 C.
